# The mediating role of self-efficacy in the relationship of obesity and physical activity among adult females

**DOI:** 10.1097/MD.0000000000046840

**Published:** 2025-12-26

**Authors:** Azza M. Atya, Abeer N. Alotaibi, Asma M. Albogami, Manar A. Takroni, Norah M. Alsaygh, Rahaf Alsaleh, Rahaf A. Husain, Afrah Almuwais, Noha F. Mahmoud

**Affiliations:** a Rehabilitation Sciences Department, Health and Rehabilitation Sciences College, Princess Nourah bint Abdulrahman University, Riyadh, Saudi Arabia.

**Keywords:** females, mediating effect, obesity, overweight, physical activity, self-efficacy

## Abstract

Obesity is a significant global public health issue. The incidence of improper physical activity (PA) has increased in recent decades. A lack of self-efficacy and the necessity to perform PA associated with excess body weight may limit its potential benefits for obese individuals. Consequently, this study investigated the mediating role of self-efficacy in the relationship between obesity and PA in adult females. In this cross-sectional study, 149 adult females aged 20 to 50 years with a body mass index >25 were recruited. The participants in this study completed a self-administered questionnaire containing the official Arabic version of the long form of the International Physical Activity Questionnaire and the General Self-Efficacy Scale. The associations between the measured variables were tested using a multiple linear regression model. This study demonstrated that obesity negatively predicted PA (*B* = −0.954, *P* < .001) and self-efficacy (*B* = −2.63, *P* < .001). When both variables were considered, obesity remained a negative predictor of PA (*B* = −1.63, *P* < .05), while self-efficacy positively predicted it (*B* = 0.963, *P* < .01). Furthermore, there were statistically significant direct and indirect associations between obesity and PA through the role of self-efficacy (*P* ≤ .05). These results indicate that self-efficacy partially mediates the effect of obesity on PA among adult females. Self-efficacy had a mediating effect on the association between obesity and PA in female adults. These findings highlight the importance of self-efficacy as a potential target for clinicians and researchers aiming to increase PA in obese populations.

**Key points**

- Obesity is a global health concern that develops because of sociocultural, environmental, psychological, metabolic, and physiological factors.
- Self-efficacy can account for differences in the adoption and maintenance of PA.
- In adult females, self-efficacy is involved in the relationship between obesity and PA. Obese adult females exhibiting diminished self-efficacy demonstrated reduced PA.

## 1. Introduction

Obesity and overweight constitute major public health concerns that have attained epidemic proportions in both poorly developing and developed countries.^[1]^ In 2020, the World Health Organization reported that the worldwide prevalence of overweight in adults aged 18 years and older was 39%. The number of obese and overweight adults is projected to reach 2.7 billion by 2025.^[2]^ A nationwide cross-sectional survey conducted in the Kingdom of Saudi Arabia revealed that the total prevalence of obesity in the Kingdom of Saudi Arabia was 35.4% compared to 31.7%, 72.0%, and 30.4% in the adjacent nations of the United Arab Emirates, Oman, and Iraq, respectively.^[3]^ Over the past couple of years, Saudi Arabia has experienced an increase in overweight and obesity among young adults, which poses a substantial risk to public health.^[4]^ The financial burden of overweight and obesity constitutes approximately 2.19% of the worldwide gross domestic product, with projections indicating an increase of 3.29% by 2060.^[5]^ Consequently, obesity has been proposed to be the 2nd most important modifiable factor contributing to preventable mortality, affecting nearly one-third of the global population.^[1]^

Considering the widespread prevalence and the significant health consequences of obesity and overweight, effective management and preventative techniques are essential. Generic public health recommendations sometimes fall short for individuals with excess weight. In practice, adults with overweight or obesity often face complex, multifactorial barriers that reduce their motivation and engagement in regular activities, making them a critical target group for health care interventions.^[6]^ In the field of health research over few decades, obesity has been classified as a general multifactorial disorder arising from sociocultural, environmental, psychological, metabolic, and physiological factors.^[7]^ Various studies have been conducted to determine whether individual differences, such as psychological characteristics, can elucidate why certain individuals engage in health-promoting activities while others abstain.^[8]^ Individual differences in cognitive processes, such as inhibitory control, may also lead some people to overeat appetizing food in the absence of hunger, whereas others can resist it.^[7,8]^ Differences in psychological factors may therefore be essential in understanding why some people develop obesity while others maintain a healthy body weight throughout their lives.^[7,9]^ Certain mental health issues, including depression, are considered to be both the cause and consequence of overweight and obesity, in part because negative emotions can lead to overeating, and the stigma associated with obesity may adversely impact one’s mental health.^[5]^

Physical activity (PA) denotes any type of bodily movement that requires energy expenditure through skeletal muscle if it is conducted adequately and consistently. It is regarded as a key factor that contributes to energy expenditure and weight control in adults.^[10]^ Discussions regarding PA have dominated health research in recent years, as they can promote healthier body weight and lower the risk of a variety of illnesses, including cardiovascular disorders and chronic illnesses, such as cancer (e.g., colon and breast), osteoporosis, and obesity. Various interventions have been indicated to lower the prevalence of obesity, with PA being the most prevalent treatment. PA counteracts diet-induced weight gain by increasing energy consumption and creating a positive energy balance.^[11]^ Furthermore, PA has a broad impact on the normal functioning of the immunological and endocrine systems, and can decrease inflammation and oxidative stress, all of which may assist people in avoiding obesity.^[12]^

Many researchers have discussed the crucial role of an individual’s beliefs and behavior in their capability to effectively participate in particular activities, especially PA, or overcome situations concerning their lives.^[13,14]^ Albasheer et al point out that self-efficacy is generally accepted as the foundation of human performance and motivation to adopt and accomplish tasks. These findings shift the focus to using self-efficacy as a facilitatory technique to control body weight.^[15]^ Within this context, self-efficacy is started to employ in many obesity prevention strategies, including those that recommend higher consumption of plant foods while minimizing high-fat and high-sucrose foods, because self-efficacy can account for differences (along with neighborhood type, income, and household type) in the adoption and preservation of healthy eating patterns and physical exercise.^[16]^ Experimental studies have shown that self-efficacy can mediate the effects of interventions on PA behavior.^[17]^ For instance, Darker et al^[18]^ discovered that participants exhibiting the most significant changes in walking self-efficacy following a single walking intervention session demonstrated the largest increase in objectively assessed walking behavior.^[18]^ A study investigated the influence of self-efficacy on behavior and weight changes, demonstrating that self-efficacy is a significant predictor of effective weight loss practices.^[16]^ Additionally, a systematic review focused on youth found that while self-efficacy is often associated with improved compliance in health behavior interventions, the direct impact of PA interventions on self-efficacy has not been extensively examined.^[19]^

This study provides distinct contributions to obesity, PA, and self-efficacy research through its stringent methodological framework and population-specific insights. In comparison with prior correlational studies, we employ formal mediation analysis based on Baron and Kenny established approach, which allows more analytical evidence than simple correlational studies and precise quantification of how much of the obesity–PA relationship operates through self-efficacy.^[20]^ Furthermore, this study covers different context-specific knowledge by focusing on Saudi adult females, a population encountering specific cultural, socioeconomic, and lifestyle barriers that may influence the established obesity–PA correlations.^[21]^ Consequently, understanding how self-efficacy functions as a mediator within these specific cultural challenges is crucial for developing effective, culturally sensitive public health interventions in the Gulf region and similar contexts. Also, increased knowledge of this relationship may lead to more comprehensive studies involving the integration of self-efficacy interventions with obesity management programs for therapists who strive to reduce or prevent obesity among adult females with poor levels of PA.

**Figure 1.**
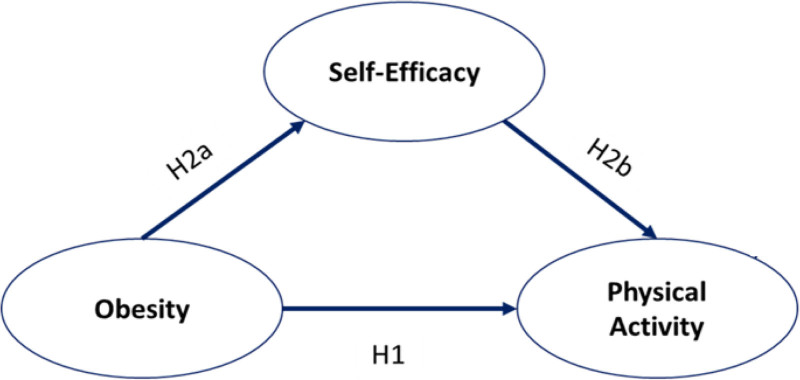
Hypothesized conceptual model.

In this study, a mediation model was constructed to examine the role of self-efficacy as a mediator of the association between obesity and PA. Age, occupation, and marital status were used as control variables in the mediating-effect test (Fig. 1). Based on this model, the following hypothesis is proposed:

**H1** There is a relationship between obesity and PA among adult females.

**H2** Self-efficacy mediates the association between obesity and PA in adult females.

**H2a** Obesity has an effect on self-efficacy among adult females.

**H2b** Self-efficacy affects PA among adult females in the presence of obesity.

## 2. Materials and methods

### 2.1. Study design

This study used a population-based, cross-sectional design. This study was approved by the Institutional Review Board of Princess Nourah bint Abdulrahman University (PNU), Riyadh (P.T. HAP-01-R-059). This study was conducted from March to April 2022. The study protocol and consent form were approved by the Health Sciences Research Center, Princess Nourah bint Abdulrahman University, before data collection.

### 2.2. Sample and participants

This study was conducted using a non-probability convenience sampling technique that focused on adult females due to both practical and contextual considerations. The sample was recruited from PNU, Riyadh, Saudi Arabia, an all-female institution in Saudi Arabia, which provided accessible participants. Furthermore, evidence suggests that women often report lower self-efficacy related to PA compared with men, and in Saudi Arabia, cultural, social, and religious factors play a significant role in shaping women’s opportunities and motivations to engage in exercise.^[21,22]^ Focusing exclusively on females, therefore, allowed us to better examine the relationship between obesity, PA, and self-efficacy within this population and to generate findings that could inform interventions tailored to women’s specific needs.

Consistent with the recognized global health challenge posed by obesity and overweight, our study focused on adults with body mass index (BMI) ≥ 25 kg/m² (overweight/obesity) to target a population at elevated obesity related health risk and to ensure alignment with the study objective and enhance internal validity of the mediation analysis. The minimum sample size was calculated using Epidemiology software Centers for Disease Control and Prevention (version 7), with a statistical power consideration that included a population size of 30,107 (based on a count of PNU students and staff according to the PNU main website), an expected frequency of 50%, an acceptable margin of error of 8%, and a design effect and cluster value of 1. The minimum required sample size was estimated to be 149 participants with a confidence level of 95%. The recruited participants were adult females between 20 and 50 years of age. The inclusion criteria were BMI ≥ 25, non-athletic female sex, absence of a high risk of a cardiac event, and ability to walk independently. Individuals who met any of the following criteria were excluded: history of orthopedic complications, cardiac dysfunction, severe renal disease, obstructive lung disease, psychiatric disease, or regular medication that would enhance or limit cardiovascular function.

### 2.3. Data collection

Participants were recruited through an Internet-based questionnaire using an online Google form that was distributed through academic emails and social media applications to both students and staff from March to April 2022. This study commenced only after receiving ethical approval from the PNU Institutional Review Board (IRB-220142). Informed consent information was inserted within the questionnaire for the participants to record their willingness to participate in the study. Proceeding past the consent form signified that each participant agreed to participate in the study. Google forms was used to avoid participant identification to ensure anonymity and to prevent potential bias during the analysis.

### 2.4. Questionnaire

The questionnaire consisted of 3 sections. The 1st gathered demographic data for each participant, including weight, height, age, educational level, occupation, marital status, and chronic systemic conditions. BMI for each participant was calculated using the individual’s height in meters and mass in kilograms, which was then used to classify them as overweight (BMI of 25.0–29.9 kg/m^2^) and obese (BMI of > 30 kg/m^2^).^[17]^ The 2nd and 3rd sections were valid and reliable scales adapted from previous studies to measure self-efficacy and PA.

Self-efficacy was assessed using the Arabic version of the General Self-Efficacy Scale (GSES).^[18,23]^ The GSES assesses a participant’s general confidence in their ability to manage challenging circumstances. It is a 10-item psychometric scale designed to assess optimistic self-beliefs in coping with various difficult demands in life. Each item was rated on a 4-point Likert scale (1 = “not at all true,” 2 = “hardly true,” 3 = “moderately true,” and 4 = “absolutely true”). The sum of all the answers was used to determine the overall self-efficacy index. The stronger the patient’s self-efficacy, the higher the overall score.^[24,25]^ The Arabic version of the GSES has shown acceptable reliability and validity in assessing self-efficacy.^[26]^

The Arabic version of the International Physical Activity Questionnaire (IPAQ), a self-administered long version, was used in this study to assess PA.^[27]^ It was developed and tested for use in adults to assess habitual PA during the preceding 7 days. The IPAQ consists of 27 items that include 4 sections of PA: occupation (7 items), transportation (6 items), household/gardening (6 items), and leisure-time activities (6 items), as well as 2 items about time spent sitting. These items track walking, as well as moderate- and vigorous-intensity activities in each domain. The total score of the IPAQ is expressed as a measure of the metabolic equivalent task (MET) minutes per week (MET-min/week). This value represents the total PA accumulated across all these domains. According to the IPAQ guidelines, PA is classified into 3 categories: low (<600 MET-min/week), moderate (600–3000 MET-min/week), and high (>3000 MET-min/week). The Arabic version of the IPAQ has demonstrated acceptable reliability and validity in assessing PA.^[28,29]^

### 2.5. Data analysis

The data collected for the current study were analyzed using SPSS version 28 (IBM, Armonk). The basic descriptive statistical data analysis for the participants is represented as mean ± standard deviation. Continuous data are presented as mean ± standard deviation, and ordinal or categorical data as counts (N) and percentages (%), unless indicated otherwise. Chi-square tests were used to examine the association between BMI and other demographic characteristics. Pearson correlation coefficient (*r*) was used to analyze the relationships between the 3 variables (obesity, self-efficacy, and obesity). The mediation effect was analyzed using Baron and Kenny mediation conceptual model.^[30]^ This model is based on multiple linear regression analysis that proposed a 3-step approach, comprising: confirming the relationship between obesity and PA by regressing the PA on obesity; confirming the relationship between obesity and self-efficacy by regressing self-efficacy on obesity; and confirming the relationship between self-efficacy and PA by regressing PA on both obesity and self-efficacy. The path from obesity to PA goes through self-efficacy (referred to as the indirect effect) and the path from obesity to PA that does not go through self-efficacy (referred to as the direct or mediator effect). Statistical significance was set at a level of .05 (95% confidence).

## 3. Results

**Figure 2.**
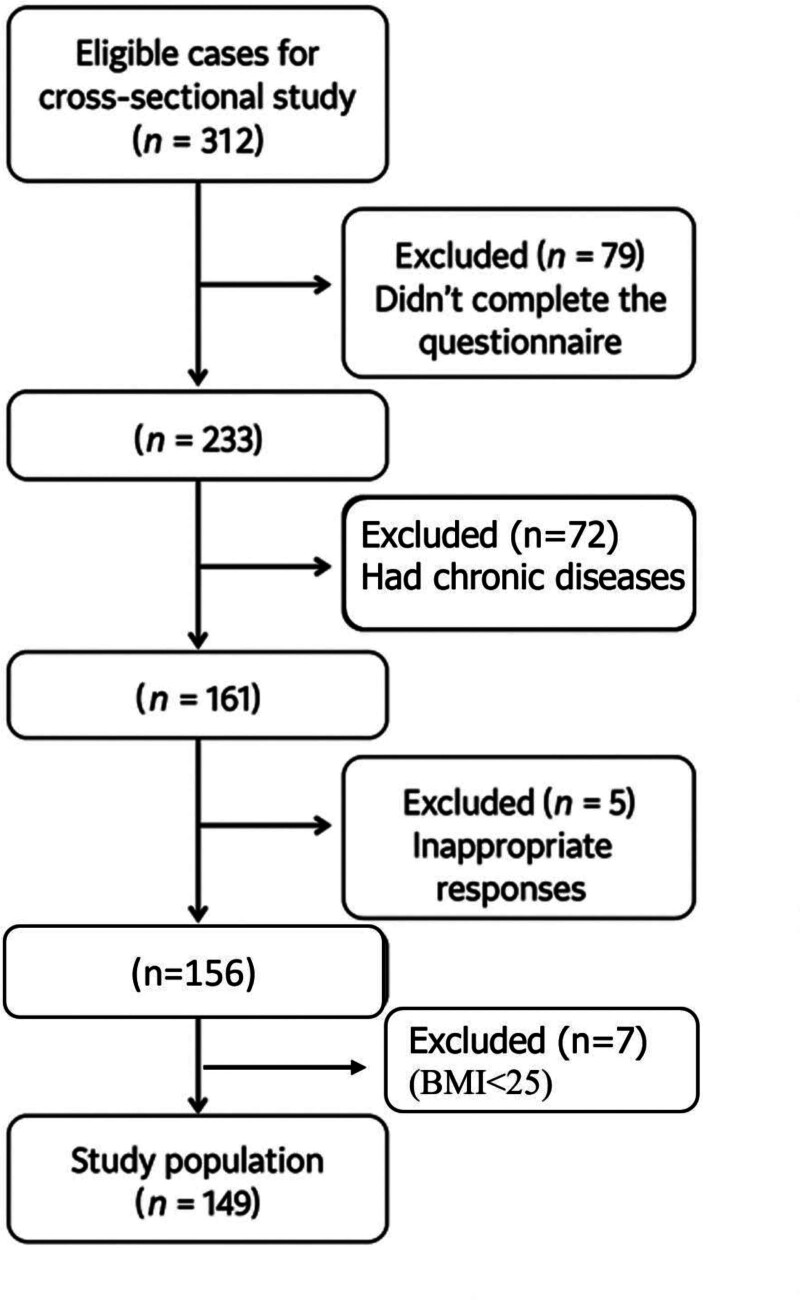
Sample size summary. BMI = body mass index.

Of the 312 adult females who accessed the questionnaire, (79) did not complete the questionnaire, (72) had chronic diseases, (5) provided improper responses, and (7) did not fit the eligibility criteria (BMI < 25). The final analysis included 149 valid questionnaires (Fig. 2).

### 3.1. Demographics

Table 1 shows the categorical demographic data of the 149 adult females who participated in the study. The data showed that 53% of the participants were aged 20 to 30 years old. Approximately 57.7% of the participants were overweight, and 42.3% were obese. Regarding marital status, 55.7% of the participants were single and 44.3% were married. In addition, 44.3% of the participants were students, and 21.5% were unemployed. Participants with a university education level or higher were the most numerous educational group (80.5%). Table 2 shows the descriptive statistics of the participants’ continuous demographic characteristics: arithmetic mean, standard deviation, and minimum and maximum of the variables for the participants in the sample. The BMI mean was 30.39 ± 4.38 kg; the mean of vigorous activity MET-min/week was 525.76 ± 1061.83; the mean of moderate activity was 339.21 ± 340.73 MET-min/week; the mean of walking activity was 305.16 ± 390.07 MET-min/week; and the mean of sitting activity was 5.5 ± 3.15 MET-min/week. Consistent with IPAQ scoring protocols, the reported MET-min/week values represent the total PA accumulated across all domains. The data confirmed that the participants’ total MET-min/week corresponded to the low PA category according to IPAQ classification.

**Table 1:** Categorical demographic characteristics of the participants (n = 149).

Demographic characteristics		N	%
BMI	Overweight	86	57.7
	Obese	63	42.3
Age	20–30 yr	79	53.0
	30–40 yr	46	30.9
	40–50 yr	24	16.1
Marital status	Single	83	55.7
	Married	66	57.7
Occupation	Student	86	42.3
	Employee	63	34.2
Educational level	High school or less	29	19.5
	University level or higher	120	80.5

BMI = body mass index.

**Table 2:** Descriptive statistics of the continuous demographic characteristics of the 149 participants.

Variables	Min.	Max.	Mean	SD
Weight (kg)	58.00	137.00	76.87	11.37
Height (cm)	145.00	174.00	159.79	5.53
BMI	24.5	51.5	30.39	4.38
Vigorous activity (MET-min/wk)	30	8640	525.76	1061.83
Moderate activity (MET-min/wk)	120	1500	339.21	340.73
Walking activity (MET-min/wk)	15	1782	305.16	390.07
Sitting (h/d)	1	18	5.50	3.15
Total PA score	15	8640	1170.13	1791.80

BMI = body mass index, MET = metabolic equivalent task, min = minutes, PA = physical activity, SD = standard deviation.

### 3.2. Associations and correlations

The associations between the participants’ BMI score classifications and categorical demographic variables (age, educational level, marital status, and occupation) were examined using the chi-square test (Table 3). The results indicated a statistically significant relation between the age range of the participants and their BMI classification (*P* = .023); most of the obese subjects engaged in the study were between 20 and 30 years of age. However, no statistically significant association was found between the educational level of participants and their BMI (*P* = .658). In addition, the data confirmed that there was a statistically significant association between the participants’ BMI classification and occupation (*P* = .001) and between the participants’ BMI and marital status (*P* = .001).

**Table 3:** Chi-square test of BMI and the participants’ categorical demographic variables.

Demographic variable	Overweight	Obese	*χ*²	*P*-value
Age			37.23	.023*
20–29	17.36	33.2		
30–39	11.63	29.4		
40–50	3.05	5.36		
Education level			15.63	.658
High school or less	20.36	16.95		
University level or higher	33.95	31.25		
Marital status			13.63	.001**
Single	2.14	2.26		
Married	1.45	2.63		
Occupation			25.45	.001**
Student	2.56	2.12		
Employee	4.26	3.56		

BMI = body mass index, *χ*² = chi-square.

* *P* < .05 is statistically significant. **P < .001 is statistically significant.

Table 4 shows the correlations between obesity, PA, and self-efficacy. The findings indicated a strong negative correlation between obesity and PA (*r* = −0.752, *P* < .01) and between obesity and self-efficacy (*r* = −0.801, *P* < .001), indicating that as obesity increased, PA and self-efficacy tended to decrease. This correlation was statistically significant, suggesting that higher levels of obesity were associated with lower PA and self-efficacy scores. In addition, a significant positive correlation was reported between self-efficacy and PA (*r* = −0.795, *P* < .01), indicating that as self-efficacy increased, so did PA.

**Table 4:** Descriptive statistics and correlations among obesity, PA, and self-efficacy.

Variable	Mean	SD	Obesity	PA	Self-efficacy
Obesity	30.39	4.38	1		
PA	2.84	0.963	−.752**	1	
Self-efficacy	28.65	0.852	−.801**	.795**	1

PA = physical activity, SD = standard deviation.

** *P* < .001 is statistically significant.

### 3.3. The mediation analysis

Mediation analysis was conducted using Baron and Kenny models,^[25]^ which depend on a series of regression analyses to test the hypothesized conceptual model (Table 5). The 1st step in this model shows the direct effect of obesity on PA. The results showed that obesity was a significant negative predictor of PA (*B* = −0.954, β ***=*** 0.303, SE = 0.684, *t* = −3.85, *P* < .001); The model explained a substantial proportion of variance in PA (*R*^2^ = 0.785, *F* = 65.25, *P* < .01) thus, the results support the hypothesis that obesity is negatively associated with PA. The 2nd step indicated that obesity had a significant negative impact on self-efficacy (*B* = −2.63, β = −0.249, SE = 0.753, *t* = −3.12, *P* < .001), and this model also explained a high proportion of the variance in self-efficacy (*R*^2^ = 0.963, *F* = 45.31, *P* < .01), providing evidence consistent with the hypothesis that higher obesity is associated with lower self-efficacy. The 3rd step explained that both obesity and self-efficacy are predictors of PA. In this model, obesity remained a significant negative predictor (*B* = −1.630, β = 0.175, SE = 0.753, *t* = −2.15, *P* < .05). At the same time, self-efficacy was a significant positive predictor (*B* = 0.963, β = 0.373, SE = 0.853, *t* = 4.85, *P* < .01). The model accounted for a large proportion of the variance in PA (*R*^2^ = 0.853, *F* = 39.84, *P* < .01) these estimates support the hypothesis that, when analyzed jointly, self-efficacy positively predicts PA while obesity remains a negative predictor. These findings indicate that self-efficacy partially mediates the relationship between obesity and PA, which verifies the main hypothesis that self-efficacy mediates the association between obesity and PA in adult females.

**Table 5:** Regression testing of the self-efficacy mediation effect.

Dependent variables	Independent variables	*B*	*β*	Std. error	*t*	*R* ^2^	*f*
PA	Constant	0.651		0.632	3.12	.785	65.25**
	Obesity	−0.954	0.303	0.684	−3.85		
PA	Constant	0.842		0.842	5.12	.853	39.84**
	Obesity	−1.630	0.175	0.753	−2.15		
	Self-Efficacy	0.963	0.373	0.853	4.85		
Self-efficacy	Constant	1.630		0.654	9.21	.963	45.31**
	Obesity	−2.630	-0.249	0.753	−3.12		

*B* = unstandardized regression coefficients, PA = physical activity, *β* = standardized regression coefficients.

** *P* < .001 is statistically significant.

**Figure 3.**
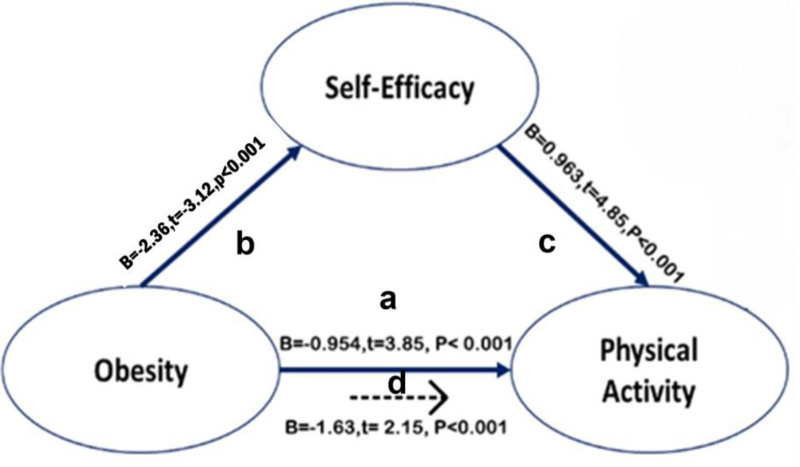
Mediating role of self-efficacy between obesity and physical activity.

Figure 3 illustrates how self-efficacy partially mediates the relationship between obesity and PA among adult females, with each path’s strength and direction shown in the diagram. The path (a) from “obesity” to “PA” represents the indirect effect of obesity on PA without controlling for self-efficacy, which shows that higher obesity is linked to lower PA. Path (b), from obesity to self-efficacy, shows that higher obesity is associated with lower self-efficacy. Path (c) from self-efficacy to “PA” indicates that higher self-efficacy leads to higher PA, and the path (d) from obesity to “PA” represents the indirect effect of obesity on PA after accounting for self-efficacy. Additionally, to ensure the validity of the regression analyses, multicollinearity was assessed using the variance inflation factor (VIF) calculations. The VIF values were 3.102 for obesity, 3.022 for PA, and 3.663 for self-efficacy, with an average VIF of 3.262. All values were substantially below the acceptable threshold of 5.0, indicating that multicollinearity did not compromise the stability or interpretability of the regression coefficients in the mediation model.

## 4. Discussion

This study investigated the role of self-efficacy in the relationship between obesity and PA among adult females. Obesity was identified as a significant inverse predictor of PA, with higher obesity levels correlating with diminished PA participation. Self-efficacy was positively correlated with PA, suggesting that individuals with diminished self-efficacy were less motivated to engage in physical exercise. Moreover, greater obesity was associated with reduced self-efficacy, which, in turn, was linked to lower PA. When self-efficacy was included in the model, the direct effect of obesity on PA was reduced but remained significant, supporting partial mediation.

The findings of this study are consistent with the data obtained in a recent meta-analysis, which supports that self-efficacy can mediate the effects of social support on PA in adolescents, indicating its potential importance across different age groups.^[31]^ Similarly, one of the systematic reviews that examined the most effective techniques in changing obese individuals’ PA self-efficacy and behavior highlighted that self-efficacy can influence dietary intake, PA, and weight change, particularly within behavioral weight loss interventions.^[17]^ Our results indicated a statistically significant negative correlation between obesity and PA among adult females, which is consistent with previous studies.^[29,32]^ These studies indicated that obese individuals had lower PA than those with normal body mass index values. Moreover, the lower PA in obese individuals may be due to the extra load from excessive body fat during weight-bearing activities.^[33]^ In addition, other studies have shown that obese individuals have reduced activity levels and consume less energy in PA than lighter individuals.^[34,35]^ O’Donovan et al examined the longitudinal association between PA levels and obesity in a cohort study encompassing 27 nations across 5 continents. The risk of obesity was approximately 30% among individuals who engaged in moderate-intensity PA and 20% lower among those who participated in high-intensity physical exercise.^[36]^ The most likely explanation for this significant negative relationship is that once obesity has been entrenched, regular PA remains incredibly low, indicating minimal energy expenditure. This indicates that a sedentary lifestyle induces obesity development, possibly resulting in a self-perpetuating vicious circle of diminished interest, reduced activity, reduced energy expenditure, and increased adiposity.^[37]^

The results of this study demonstrated that obesity and self-efficacy were negatively correlated, which has been confirmed by some existing studies.^[19,38–40]^ They revealed that self-efficacy was the most significant contributor in predicting behavioral intention among obese people, and this needs to be considered when designing nutritional interventions for this population. Additionally, the current research findings demonstrated that a lower BMI is linked to increased self-efficacy levels, which may be related to some concerns in the subject’s behaviors, such as healthy eating habits and regular exercise practice.^[41]^ Therefore, obese people have lower self-efficacy when it comes to health-related behaviors. In adults, perceived challenges in obesity management are correlated with depressed motivation and perception of one’s ability to cope with being overweight or obese.^[42]^

Additionally, the results suggested a positive correlation between self-efficacy and PA. This was supported by the work of Liou and Kulik,^[43]^ which considers self-efficacy as one of the main psychosocial determinants of obese participants’ attitudes toward PA. Therefore, residential obesity treatment programs that meet the requirements of autonomy, relatedness, and competence are likely to increase self-efficacy regarding PA.^[43]^ This agrees with the work of Medrano et al,^[39]^ which indicated that self-efficacy is one of the most powerful predictors of PA, as it affects one’s personal motivation to practice PA. In accordance with these findings, obese subjects maintained high efficacy beliefs about overcoming barriers to PA and those inherent in their environment. In addition, adherence to exercise programs to promote cardiopulmonary fitness may have a significant influence on the quality of life and self-efficacy, thus promoting PA maintenance. However, the relationship between PA and its cognitive determinants needs to be further examined using randomized controlled trials of PA interventions.^[39]^

This study revealed strong interconnections among obesity, PA, and self-efficacy, as indicated by elevated *R*² values (e.g., 0.785, 0.853, 0.963) in our mediation model. Although these values may seem high in behavioral research, these values reflect the inherent strength of the relationships among the measured variables, which are theoretically well-established and closely interconnected in the literature.^[17,32,43]^ Moreover, the utilization of a relatively homogeneous sample and highly reliable measurement instruments in this study reduced unexplained variance and enhanced model explanatory power. Conversely, the risk of overfitting is considered minimal due to the model’s simplicity and solid theoretical foundation, thereby affirming that the elevated *R*² values signify authentic and strong association within this specific population.

The findings of this study are consistent with those of Sunarti et al. This study revealed that women with higher self-efficacy or confidence in their capabilities were more inclined to participate in regular physical activities. This emphasizes that different forms of activity affect women’s self-efficacy in different ways. Resistance-based activities were identified as highly empowering, enhancing participants’ confidence in their capacity to exercise, and impacting their mental and emotional well-being. These exercises empowered women to gain greater control over their physical capabilities and enhanced their motivation to participate in more regular exercise. In contrast, cardiorespiratory workouts, such as jogging and swimming, similarly improved self-efficacy beliefs; however, the feelings of empowerment, achievement, and self-worth were more significant with resistance training. This indicates that the nature of exercise significantly influences the enhancement of self-efficacy, particularly in activities that foster a sense of strength.^[44]^ Recent research has shown that self-efficacy in nutrition and PA is a crucial psychological determinant affecting behavioral change and is a vital focus for interventions designed to enhance general health, PA, and nutritional consumption. In addition, enhancements in self-efficacy after dietary intervention were associated with more significant weight reduction and physical performance.^[45]^

Despite the well-established inverse relationship between obesity and physical inactivity. Our study clarifies the underlying mechanisms of this association among adult Saudi females. By demonstrating that self-efficacy partially mediates the effect of obesity on PA, the findings emphasize the substantial influence of obese people’s beliefs in their own capabilities. This profound understanding advances the existing knowledge by highlighting self-efficacy as a modifiable psychological pathway that can be effectively enhanced through evidence-based techniques. Focusing on self-efficacy as a key mediator provides novel and actionable insights for healthcare practitioners to integrate the self-efficacy stimulating elements as behavioral counseling, motivational interviewing, and exercise education, into obesity management and PA promotion initiatives to empower individuals to overcome perceived barriers and sustain healthy behaviors.^[17,39]^

Our study demonstrated the mediating role of self-efficacy in the relationship between obesity and PA among adult females and may be of value in developing interventions to promote PA in this population. However, this study has several limitations. Firstly, the cross-sectional design prevents the establishment of causal relationships between obesity, self-efficacy, and PA; future research utilizing a longitudinal design could address this limitation and confirm the directionality of these relationships. Second, this study focused solely on the mediating role of self-efficacy, and subsequent research should consider other potential mediators, such as social support, perceived barriers to exercise, and access to fitness facilities, to provide a more comprehensive understanding of other factors influencing PA in obese adult females. Third, from a methodological perspective, only age, occupation, and marital status were included as covariates in the mediation model; other sociodemographic factors as education, were described but not analyzed. This prespecified adjustment reflected data and model-stability considerations, but may leave residual confounding; therefore, direct and indirect effects are interpreted with appropriate caution. Future studies should include a broader sociodemographic set and sensitivity analyses to further test the resilience of the mediation pathways. Lastly, only adult females were included in this study; additional research is needed to ascertain whether these findings are generalizable in males and across age groups.

## 5. Conclusion

This study establishes that self-efficacy partially mediates the relationship between PA and obesity among adult females. Obesity directly and indirectly contributes to mitigating PA, while self-efficacy enhances individuals’ engagement in PA, indirectly influencing obesity outcomes. These findings emphasize the importance of incorporating psychological factors such as self-efficacy into obesity intervention programs in adult females to improve PA adherence and promote better weight management. Future research should further examine other potential mediators to develop more comprehensive strategies for obesity.

## Acknowledgments

The authors acknowledge the support provided by Princess Nourah bint Abdulrahman University Researchers Supporting Project Number PNURSP2025R206, Princess Nourah bint Abdulrahman University, Riyadh, Saudi Arabia.

## Author contributions

**Conceptualization:** Azza M. Atya, Abeer N. Alotaibi, Asma M. Albogami, Manar A. Takroni, Norah M. Alsaygh, Rahaf Alsaleh, Rahaf A. Husain, Noha F. Mahmoud.

**Funding acquisition:** Noha F. Mahmoud.

**Investigation:** Abeer N. Alotaibi, Asma M. Albogami, Manar A. Takroni, Norah M. Alsaygh, Rahaf Alsaleh, Rahaf A. Husain.

**Methodology:** Abeer N. Alotaibi, Asma M. Albogami, Manar A. Takroni, Norah M. Alsaygh, Rahaf Alsaleh, Rahaf A. Husain.

**Supervision:** Azza M. Atya.

**Validation:** Afrah Almuwais, Noha F. Mahmoud.

**Writing – original draft:** Azza M. Atya, Abeer N. Alotaibi, Norah M. Alsaygh, Rahaf Alsaleh, Rahaf A. Husain, Afrah Almuwais.

**Writing – review & editing:** Azza M. Atya, Noha F. Mahmoud.
